# Long-term health-related quality of life following an online rehabilitation program after total laryngectomy: a 3-year retrospective longitudinal study

**DOI:** 10.25122/jml-2026-0060

**Published:** 2026-06

**Authors:** Paula Luiza Bejenaru, Gloria Simona Berteșteanu, Catrinel Beatrice Simion-Antonie, Ruxandra Ioana Nedelcu-Stancalie, Teodora Elena Schipor-Diaconu, Simona Andreea Rujan, Bianca Petra Taher, Raluca Grigore, Bogdan Popescu, Irina Doinița Popescu, Alexandru Nicolaescu, Anca Ionela Cîrstea, Șerban Gabriel Vifor Berteșteanu

**Affiliations:** 1 Department 12 – Otorhinolaryngology, Ophthalmology, Faculty of Medicine, Carol Davila University of Medicine and Pharmacy, Bucharest, Romania; 2 Otorhinolaryngology Department, Dr. Carol Davila Central Military Emergency University Hospital, Bucharest, Romania; 3 Otorhinolaryngology Department, Coltea Clinical Hospital, Bucharest, Romania; 4 Otorhinolaryngology Department, Prof. Dr. Dimitrie Gerota Emergency Hospital, Bucharest, Romania; 5 Otorhinolaryngology Department, University Emergency Hospital Bucharest, Bucharest, Romania

**Keywords:** total laryngectomy, quality of life, online rehabilitation, TEVP, EORTC QLQ-C30, tele-rehabilitation

## Abstract

This study aimed to evaluate health-related quality of life (HRQoL) trajectories after participation in an online rehabilitation course. A retrospective observational longitudinal study was conducted in accordance with the STROBE (Strengthening the Reporting of Observational Studies in Epidemiology) guidelines. We analyzed 48 patients who completed a multimodal online rehabilitation course in 2022. The curriculum covered anatomical foundations and practical techniques for voice production, deglutition, respiration, and olfactory recovery. HRQoL was assessed using the EORTC QLQ-C30 instrument at baseline (T0) and annually for 3 years (T1, T2, T3). All scores were linearly transformed to a 0–100 scale. Five patients were censored due to mortality during follow-up. Longitudinal exploratory analyses were performed using repeated-measures comparisons where appropriate. Among the 43 survivors at 3 years, clinically relevant longitudinal improvements were observed in global health status/QoL (from 58.4 ± 18.2 to 85.7 ± 9.8, +27.3 points), physical functioning (+20.9 points), role functioning (+22.5 points), and social functioning (+19.5 points). Symptom burden decreased markedly, particularly fatigue (−22.5 points), dyspnea (−22.7 points), and pain (−17.3 points). Patients with TEVP (tracheoesophageal voice prosthesis) complications (*n* = 3), defined as an exploratory group, tended to have less favorable recovery trajectories and lower global QoL scores at 3 years compared to those without complications (*P* = 0.012). The most pronounced improvements occurred within the first post-intervention year and remained stable thereafter. Patients participating in the online rehabilitation program demonstrated sustained improvements in HRQoL over the follow-up period; however, the absence of a control group precludes causal inference.

## Introduction

Laryngeal cancer remains a significant public health challenge in Romania, which exhibits some of the highest incidence rates in the European Union, particularly among the male population [1]. Recent epidemiological data for Romania indicate an annual burden of over 1,800 new laryngeal malignancies and 1,088 deaths, with an age-standardized incidence rate that ranks it among the most affected European nations [1, 2].

Total laryngectomy remains the gold standard curative treatment for locally advanced laryngeal carcinoma. While providing excellent oncological control, the procedure results in the permanent loss of the natural voice and significant alterations in breathing, deglutition, and olfaction. These changes profoundly impact patients’ physical, psychological, and social functioning, thereby reducing their health-related quality of life (HRQoL).

The COVID-19 pandemic disrupted traditional in-person rehabilitation and highlighted the limitations of conventional care models. Conversely, it demonstrated the potential of digital health solutions to maintain continuity of care. In the current post-pandemic period, online rehabilitation programs have emerged as flexible, accessible tools that bridge the gap between healthcare providers and patients, especially those facing geographical or logistical barriers.

In 2022, a structured online rehabilitation course was organized to address these needs, covering anatomical foundations, social and healthcare guidance, and practical techniques to improve functional outcomes. This study evaluates the 3-year impact of this digital intervention on the QoL of laryngectomized patients.

## Material and Methods

### Study design

This retrospective observational longitudinal study included 48 patients enrolled in an online rehabilitation course following total laryngectomy. Assessments were conducted at four time points: T0 (baseline, defined as the assessment performed immediately before participation in the online rehabilitation course), T1 (1 year), T2 (2 years), and T3 (3 years). Five patients died during the follow-up period and were excluded from longitudinal analyses post-mortem. This study follows the Strengthening the Reporting of Observational Studies in Epidemiology (STROBE) guidelines. The curriculum integrated multimodal rehabilitation aspects: anatomical education, vocal production (tracheoesophageal voice prosthesis [TEVP] optimization), stoma care, pulmonary rehabilitation, and sensory recovery (olfactory and gustatory function).

### Participants

#### Inclusion criteria

Eligible participants were patients who underwent total laryngectomy for laryngeal cancer with primary tracheoesophageal puncture (TEP). Participants were within the first year following treatment at baseline (T0), had completed the European Organization for Research and Treatment of Cancer Quality of Life Questionnaire-Core 30 (EORTC QLQ-C30) at baseline, and had available follow-up data.

**Figure 1:**
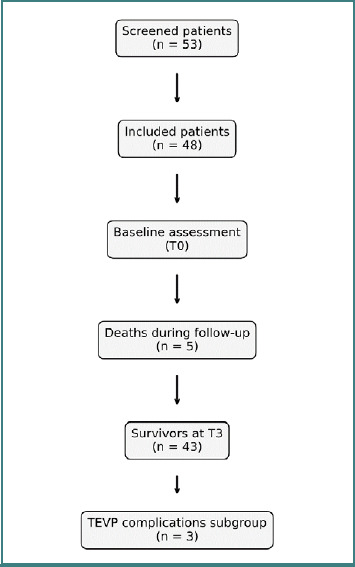
Patient flow diagram of the retrospective longitudinal cohort. Flowchart illustrating patient selection, follow-up, mortality during follow-up, and subgroup allocation for TEVP-related complications.

The patient selection and follow-up process are summarized in Figure 1.

#### Assessment tool

Quality of life was measured using the European Organization for Research and Treatment of Cancer Quality of Life Questionnaire – Core 30 (EORTC QLQ-C30), version 3.0. This validated instrument consists of 30 items and evaluates:

- Five functional scales (FS): physical, role, emotional, cognitive, and social functioning;
- Three symptom scales: fatigue, pain, and nausea/vomiting;
- Six single-item symptoms: dyspnea, insomnia, appetite loss, constipation, diarrhea, and financial difficulties;
- Two global items assessing overall health status and quality of life (global health status / QoL).

Most items are scored on a 4-point Likert scale (1 = ‘not at all’ to 4 = ‘very much’), while the two global items are scored on a 7-point scale.

### Scoring procedure

Raw scores were linearly transformed into standardized scores ranging from 0 to 100, according to the official EORTC QLQ-C30 scoring manual [3]. For functional scales and global health status/QoL, higher scores represent better functioning or better quality of life. For symptom scales and items, higher scores indicate greater symptom burden.

Scores were calculated only when patients answered at least half of the items within a scale, following EORTC recommendations. Missing data were handled according to the EORTC guidelines.

### Statistical analysis

Data analysis was performed using Python (v3.14.2; Python Software Foundation) with the SciPy and Pandas libraries and Microsoft Excel 2016. Descriptive statistics included means and standard deviations (SD). Longitudinal exploratory analyses were performed using paired longitudinal comparisons and, where appropriate, exploratory repeated-measures analyses. A change of ≥10 points was defined as the minimal clinically important difference (MCID).

### Online rehabilitation course structure

The online rehabilitation program was delivered in a single day and lasted approximately 8 hours. The educational sessions were conducted by ENT specialists and ENT residents with experience in the management and rehabilitation of laryngectomized patients.

The curriculum included several major topics relevant to postoperative adaptation following total laryngectomy, including:

- anatomy before and after total laryngectomy;
- tracheostomy care and hygiene principles;
- voice rehabilitation using a tracheoesophageal prosthesis;
- pulmonary rehabilitation;
- rehabilitation of smell and taste;
- swallowing rehabilitation;
- management of postoperative and prosthesis-related emergencies.

The course was conducted online via Zoom and incorporated slide presentations, educational videos, and live practical demonstrations. After each educational section, participants were allowed to ask questions and discuss difficulties or uncertainties related to postoperative rehabilitation.

Quality-of-life questionnaires (EORTC QLQ-C30) were completed online using Google Forms before participation in the course (T0), and subsequently at 1 year (T1), 2 years (T2), and 3 years (T3). Data collection procedures followed the ethical principles and requirements described previously.

This educational intervention was intended to provide structured informational support and facilitate long-term postoperative adaptation in patients undergoing total laryngectomy and tracheoesophageal voice rehabilitation.

## Results

A total of 48 patients who participated in an online rehabilitation program following total laryngectomy were included in this observational longitudinal retrospective study. Health-related quality of life (HRQoL) was evaluated using the EORTC QLQ-C30 questionnaire at baseline (T0) and subsequently at 1 year (T1), 2 years (T2), and 3 years (T3).

All patients were diagnosed with stage IVa laryngeal carcinoma and underwent total laryngectomy with bilateral neck dissection and primary tracheoesophageal puncture with TEVP placement. In all cases, the TEVP used was Provox Vega.

During the follow-up period, five patients (10.4%) died and were censored from longitudinal analyses after the time of death. In addition, three patients (6.25%) developed TEVP-related complications. Due to the distinct clinical evolution observed in these patients, they were analyzed separately in a subgroup analysis.

The study cohort consisted predominantly of male patients with a history of tobacco exposure. Most patients were from urban areas and had secondary education (Table 1).

**Table 1:** Baseline characteristics at T0

Variable	Value
Age, mean ± SD (years)	64.2 ± 8.1
Age range (years)	50–79
Sex – Male, *n* (%)	45 (93.7%)
Smoking – Yes, *n* (%)	48 (100%)
Alcohol consumption – Yes, *n* (%)	35 (72.9%)
TNM stage	IVa in all patients
Occupational toxic exposure – Yes, *n* (%)	33 (68.7%)
Adjuvant therapy before surgery – Radiotherapy, *n* (%)	15 (31.2%)
Adjuvant therapy before surgery – Chemotherapy, *n* (%)	4 (8.3%)
No adjuvant therapy before surgery, *n* (%)	29 (60.5%)
Charlson Comorbidity Index (CCI), mean ± SD	4.1 ± 1.2
Education – Primary school, *n* (%)	15 (31.2%)
Education – Secondary/High school, *n* (%)	26 (54.2%)
Education – University level, *n* (%)	7 (14.6%)
Residence – Urban, *n* (%)	31 (64.6%)
Residence – Rural, *n* (%)	17 (35.4%)

### Evolution of quality of life over time (survivors, *n* = 43 at T3)

The mean scores for the main EORTC QLQ-C30 scales across the four time points are presented in Table 2.

**Table 2:** Mean scores (± SD) of EORTC QLQ-C30 scales at baseline and during follow-up (0–100 transformed scores)

Scale / Item	T0 (Baseline) Mean ± SD	T1 (1 year) Mean ± SD	T2 (2 years) Mean ± SD	T3 (3 years) Mean ± SD	Change* (T0–T3)
Global health status / QoL	58.4 ± 18.2	72.6 ± 14.1	81.3 ± 11.7	85.7 ± 9.8	+27.3
Physical functioning	68.2 ± 16.5	78.9 ± 12.4	86.4 ± 9.3	89.1 ± 7.6	+20.9
Role functioning	65.7 ± 19.3	76.8 ± 15.2	84.9 ± 11.5	88.2 ± 8.9	+22.5
Emotional functioning	71.3 ± 14.8	79.5 ± 12.6	84.7 ± 10.2	87.4 ± 8.1	+16.1
Cognitive functioning	78.6 ± 13.1	83.2 ± 11.4	87.1 ± 9.8	89.5 ± 7.4	+10.9
Social functioning	67.4 ± 17.9	76.1 ± 14.3	83.6 ± 12.1	86.9 ± 9.5	+19.5
Fatigue	41.2 ± 16.7	29.8 ± 14.5	22.4 ± 12.3	18.7 ± 10.6	-22.5
Dyspnea	38.9 ± 21.4	26.3 ± 17.8	19.5 ± 13.9	16.2 ± 11.4	-22.7
Pain	29.6 ± 15.2	21.4 ± 13.1	15.8 ± 10.7	12.3 ± 9.1	-17.3
Insomnia	35.7 ± 18.3	26.1 ± 15.9	19.4 ± 12.8	16.8 ± 11.2	-18.9
Appetite loss	24.8 ± 14.6	16.9 ± 12.3	11.2 ± 9.5	8.7 ± 7.9	-16.1
Financial difficulties	22.4 ± 19.1	18.7 ± 16.4	14.3 ± 13.2	12.6 ± 11.8	-9.8

A progressive improvement in HRQoL scores was observed over the 3-year follow-up period. The most notable changes were observed between baseline and the first postoperative year, while subsequent improvements appeared more gradual.

Global health status/QoL increased from 58.4 ± 18.2 at baseline to 85.7 ± 9.8 at T3. Similar longitudinal trends were observed in all major functional domains, including physical, role, emotional, cognitive, and social functioning.

Among the symptom scales, fatigue, dyspnea, pain, and insomnia showed progressive reductions throughout the follow-up period. Dyspnea and fatigue showed the largest absolute reductions between T0 and T3.

According to EORTC interpretation criteria, most observed longitudinal changes exceeded the 10-point threshold generally considered clinically meaningful.

**Figure 2:**
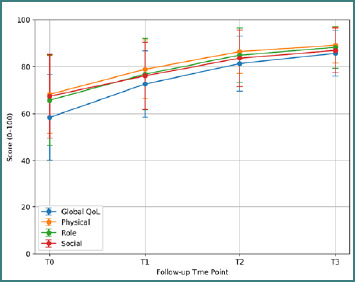
Evolution of functional scales and global health status/QoL during follow-up. Line plots illustrating longitudinal changes in EORTC QLQ-C30 functional scales and global health status/QoL scores at baseline (T0), 1 year (T1), 2 years (T2), and 3 years (T3). Error bars represent standard deviations (SD). Higher scores indicate better functioning and quality of life.

**Figure 3:**
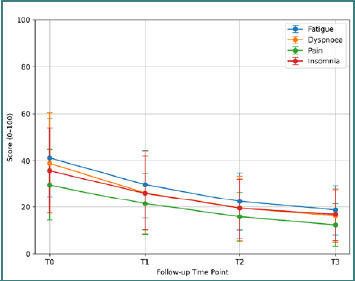
Evolution of symptom scales during follow-up. Line plots illustrating changes in EORTC QLQ-C30 symptom scales across the four assessment time points (T0–T3). Error bars represent standard deviations (SD). Higher scores indicate greater symptom burden.

Figure 2 illustrates the evolution of functional scales and global QoL during follow-up, while Figure 3 summarizes the temporal evolution of symptom scales.

### Subgroup analysis: patients with TEVP complications

At T3, patients who experienced TEVP-related complications (*n* = 3) had lower HRQoL scores than those without prosthesis-related complications (*n* = 40).

Global health status/QoL scores were 74.3 ± 8.1 in the complication subgroup versus 86.8 ± 9.2 in the non-complication subgroup (*P* = 0.012). Physical functioning scores were 79.4 ± 6.5 versus 90.2 ± 7.1 (*P* = 0.008), while dyspnea scores were higher in patients with complications (31.7 ± 12.4 vs. 14.8 ± 10.2; *P* = 0.019). Social functioning scores were also lower in patients with complications (76.5 ± 9.3 vs. 88.1 ± 8.7; *P* = 0.031).

These findings suggest a less favorable recovery trajectory among patients who developed prosthesis-related complications, particularly regarding respiratory symptoms and social reintegration. However, because only three patients presented with TEVP-related complications, these findings should be interpreted with caution and considered exploratory.

**Figure 4:**
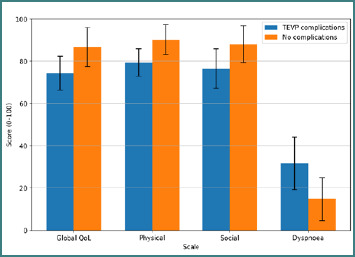
HRQoL outcomes at T3 according to TEVP complication status. Bar chart comparing EORTC QLQ-C30 scores at 3 years between patients with TEVP-related complications (*n* = 3) and those without complications (*n* = 40). Error bars represent standard deviations (SD).

Patients with TEVP-related complications also demonstrated lower scores in global QoL, physical functioning, role functioning, and social functioning, together with higher dyspnea scores at 3 years (Figure 4).

Within the observed cohort (*n* = 43), longitudinal analyses demonstrated statistically significant changes in global health status/QoL over time (mean increase of 27.3 points; *P* < 0.001), accompanied by reductions in symptom burden, particularly fatigue and dyspnea.

Dyspnea showed the greatest improvement among symptom scales, with larger reductions observed in patients without prosthesis-related complications than in those who experienced complications (*P* = 0.023).

Overall, the observed longitudinal trends suggest gradual improvement in HRQoL and functional recovery over the 3-year postoperative period among surviving laryngectomized patients who participated in the rehabilitation program. Due to the retrospective observational design of the study, no causal relationship between participation in the online course and HRQoL outcomes can be established.

## Discussion

This retrospective longitudinal study evaluated the evolution of HRQoL over a 3-year follow-up period in 48 patients who participated in a structured online rehabilitation program after total laryngectomy. Using the EORTC QLQ-C30 questionnaire, progressive longitudinal improvements were observed across multiple functional and symptom domains during follow-up. Mean global health status/QoL scores increased from 58.4 ± 18.2 at baseline to 85.7 ± 9.8 at 3 years, corresponding to a mean change of +27.3 points and exceeding the minimal clinically important difference (MCID) commonly reported for head and neck cancer populations. Similar clinically relevant longitudinal changes were observed in physical functioning (+20.9 points), role functioning (+22.5 points), and social functioning (+19.5 points), accompanied by reductions in fatigue (−22.5 points), dyspnea (−22.7 points), pain (−17.3 points), and insomnia (−18.9 points).

The observed recovery pattern, characterized by more pronounced improvements during the first postoperative year followed by slower but persistent gains over time, is generally consistent with previous reports evaluating rehabilitation after total laryngectomy [4-7]. Souza *et al*. reported that HRQoL in laryngectomized patients frequently declines during active oncologic treatment and subsequently stabilizes or improves within the first postoperative year, particularly when functional vocal rehabilitation is achieved [4]. In their cohort, patients who were rehabilitated with TEVP demonstrated more favorable QoL outcomes than those using an electrolarynx or esophageal speech [4].

Our findings are also consistent with the potential contribution of structured rehabilitation strategies in post-laryngectomy adaptation [5-7]. The online rehabilitation curriculum included anatomical education, tracheostomy care, principles of breathing and swallowing rehabilitation, voice restoration techniques, and olfactory rehabilitation. Favorable longitudinal changes in both functional scales and symptom burden were observed among participants throughout the follow-up period. Similarly, Massaro *et al*. reported that TEVP-based vocal restoration may facilitate recovery of communication abilities and contribute to satisfactory long-term quality-of-life outcomes in laryngectomized patients [7]. Their study additionally highlighted the importance of effective communication restoration as a relevant component of psychosocial reintegration and long-term adaptation [7].

An additional finding of the present study was the less favorable HRQoL trajectory observed in the small subgroup of patients who developed TEVP-related complications. At 3 years, these patients presented lower scores in global QoL, physical functioning, role functioning, and social functioning, together with persistently higher dyspnea scores. However, because only three patients experienced TEVP-related complications, these subgroup analyses should be considered exploratory and interpreted cautiously. Device-related complications, including prosthesis leakage and granulation tissue formation, may interfere with respiratory comfort, communication, and social reintegration, potentially influencing postoperative adaptation trajectories. These observations are consistent with previous reports suggesting that prosthesis-related complications may adversely affect patient-reported outcomes, even when long-term voice restoration remains functionally satisfactory [8-10]. Maniaci *et al*. similarly emphasized that although TEVP generally yields favorable voice-related quality-of-life outcomes, prosthesis-related complications remain an important clinical challenge that requires timely management and follow-up [11].

Vlachtsis *et al*. reported that, despite persistent impairments in selected domains such as speech, social interaction, and physical symptoms, overall quality of life after total laryngectomy may reach satisfactory levels with adequate rehabilitation support [12]. The longitudinal trends observed in our cohort are generally consistent with these observations, as most surviving patients achieved relatively high global health status/QoL scores by the third year of follow-up.

The online format of the rehabilitation program also deserves consideration. While most available studies have focused on in-person rehabilitation models, digitally delivered supportive care approaches may represent a feasible complementary option for selected laryngectomized patients, particularly for those facing geographic limitations, mobility difficulties, or barriers to repeated hospital-based follow-up. Nevertheless, due to the observational design of the present study and the absence of a comparator group, no direct conclusions regarding the efficacy of online rehabilitation compared to conventional rehabilitation approaches can be established.

### Strengths and limitations

Strengths of this single-center retrospective study include the longitudinal design with assessments up to 3 years, the use of a validated international instrument (EORTC QLQ-C30), and the transformation of scores to the standardized 0–100 scale, allowing direct comparison with the broader literature. The inclusion of a subgroup analysis for TEVP complications provides additional clinical insight. Still, because only three patients experienced TEVP-related complications, subgroup analyses should be interpreted as exploratory and descriptive.

A major strength of this study is the 3-year longitudinal follow-up, which provides descriptive long-term observational data regarding digitally delivered rehabilitation support after laryngectomy. While many digital health initiatives were implemented as temporary emergency measures, our data suggest that favorable longitudinal trends of a structured online course are not merely transient. The persistence of the observed longitudinal HRQoL trends over 36 months supports further prospective controlled evaluation of structured digital rehabilitation strategies in laryngectomized patients.

However, several limitations must be acknowledged. First, the absence of a control group represents the principal methodological limitation and precludes causal attribution of the observed HRQoL improvements to the online rehabilitation program itself. Natural postoperative recovery, routine clinical follow-up, patient motivation, survivorship effects, and prosthesis-related factors may all have contributed to the favorable longitudinal trajectories observed. Nevertheless, the extended 3-year follow-up still provides clinically relevant descriptive information on the evolution of HRQoL among patients participating in structured online rehabilitation.

Second, the study relied exclusively on the EORTC QLQ-C30 questionnaire and did not include head and neck-specific instruments such as the EORTC QLQ-H&N35 or dedicated voice-related measures, including the Voice Handicap Index (VHI) and Voice-Related Quality of Life (V-RQOL) scale. The absence of these disease-specific tools limited detailed assessment of speech intelligibility, swallowing dysfunction, olfactory impairment, and voice-related outcomes.

Third, several retrospective design-related limitations should be considered. Detailed oncologic treatment timelines, salvage surgery status, and certain treatment-related variables were not consistently available in the dataset. In addition, the small subgroup of patients with TEVP-related complications (*n* = 3) substantially limited statistical power for subgroup comparisons; therefore, these analyses should be interpreted as exploratory and descriptive.

Furthermore, because patients who died during follow-up could not contribute to later HRQoL assessments, survivorship bias may have contributed to overestimation of long-term HRQoL outcomes. Finally, both the rehabilitation course and questionnaire administration were conducted online. Participation, therefore, required internet access and a minimum level of digital literacy, which may have introduced selection bias and limited the generalizability of the findings.

Future prospective, multicenter studies should combine general and disease-specific QoL instruments with voice-specific measures and include larger samples to evaluate better the impact of complications and the effectiveness of digital versus traditional rehabilitation programs. Long-term follow-up beyond 3 years would also be valuable to assess the durability of the observed improvements.

## Conclusion

In conclusion, favorable long-term HRQoL trajectories were observed over the three-year follow-up period among patients participating in a structured online rehabilitation program after total laryngectomy. Improvements were noted across multiple functional domains, accompanied by reductions in symptom burden during longitudinal follow-up. However, given the retrospective observational design and the absence of a comparator group, these findings should be interpreted cautiously and cannot establish a causal relationship between participation in the online course and the observed outcomes.

Patients who developed TEVP-related complications appeared to have less favorable recovery patterns in selected HRQoL domains, particularly physical functioning, dyspnea, and social functioning. However, these subgroup analyses remain exploratory given the limited sample size.

Overall, the present findings support further prospective controlled investigation of structured multidisciplinary rehabilitation strategies, including digitally delivered supportive care approaches, in post-laryngectomy survivorship management.
